# Applications of different forms of nitrogen fertilizers affect soil bacterial community but not core ARGs profile

**DOI:** 10.3389/fmicb.2024.1447782

**Published:** 2024-10-01

**Authors:** Ruiqiang You, Yang Yu, Min Shen, Yanzhou Zhang, Jian Hong, Yijun Kang

**Affiliations:** ^1^Jiangsu Key Laboratory for Bioresources of Saline Soils, Yancheng Teachers University, Yancheng, Jiangsu, China; ^2^College of Environmental Science and Engineering, Yangzhou University, Yangzhou, China

**Keywords:** antibiotics resistance genes, bacterial community, chemical nitrogen fertilizer, soil metagenomic sequencing, core resistome

## Abstract

The objective of this study was to investigate the impact of various chemical nitrogen fertilizers on the profile of antibiotic resistance genes (ARGs) in soil. A microcosm experiment was conducted with four treatments, including CK (control with no nitrogen), AN (ammonium nitrogen), NN (nitrate nitrogen), and ON (urea nitrogen), and the abundance of ARGs was assessed over a 30-day period using a metagenomic sequencing approach. The levels of core ARGs varied between 0.16 and 0.22 copies per cell across different treatments over time. The abundance of core ARGs in the ON treatment closely resembled that of the CK treatment, suggesting that environmentally friendly nitrogen fertilizers, particularly those in controlled release formulations, may be preferable. The core ARG abundance in the AN and NN treatments exhibited noticeable fluctuations over time. Overall, chemical nitrogen fertilizers had minimal effects on the core ARG profile as determined by principal component analysis and clustering analyses. Conversely, distinct and significant changes in bacterial communities were observed with the use of different nitrogen fertilizers. However, the influence of nitrogen fertilizers on the core ARGs is limited due to the unaffected potential bacterial hosts. Nitrogen-cycling-related genes (NCRGs), such as those involved in nitrogen-fixing (*nifK*, *nifD*, *nifH*) and denitrification (*narG*, *napA*, *nirK*, *norB*, *nosZ*) processes, exhibit a positive correlation with ARGs (*rosA*, *mexF*, *bacA*, *vanS*), indicating a potential risk of ARG proliferation during intense denitrification activities. This study indicates that the application of chemical nitrogen has a minimal effect on the abundance of ARGs in soil, thereby alleviating concerns regarding the potential accumulation of ARGs due to the use of chemical nitrogen fertilizers.

## Introduction

The annual consumption of antibiotics in China reaches millions of kilograms (Zhang et al., 2015), with a significant portion being excreted into the environment by animals due to incomplete absorption and metabolism (Daghrir and Drogui, 2013). This phenomenon has led to the proliferation of antimicrobial resistance, driven by antibiotic resistance genes (ARGs) as a result of the improper use or excessive administration of antibiotics. The World Health Organization has identified this issue as a major threat to human health in the current century (Edwards et al., 2021). Despite composting and high temperature treatment, certain antibiotics and ARGs retain their biological activity upon entering the environment and can become more prevalent in soil through horizontal gene transfer (HGT) (Su et al., 2015; Wang et al., 2015; Kang et al., 2016), thereby contributing to the global dissemination of ARGs.

Agricultural soil serves as a significant locus for the occurrence and dissemination of ARGs due to the substantial influx of manure (Kang et al., 2018b; Hilaire et al., 2022), microplastic residues (Zhu et al., 2022), and the application of pesticides and hormones (García et al., 2020). Of these factors, fertilization emerges as paramount, given its role in modulating soil microbial compositions (Zeng et al., 2016; Shawver et al., 2021) and introducing heavy metals and biologically active compounds into the soil (Guo et al., 2018). There are persistent debates regarding the consequences of fertilization practices. Overall, the temporary application of composted manure may induce the proliferation of ARGs in the topsoil layer (Xie et al., 2018a; Kang et al., 2022), whereas prolonged use of manure may enhance the variety of ARGs without affecting their quantities (Wang F. et al., 2018). Nevertheless, research has indicated that the utilization of manure from cattle treated with antibiotics can have lasting effects on the soil resistome and microbial community structure for a minimum of 3 years (Shawver et al., 2021).

Controversies surrounding chemical fertilizers have been attributed to the increased indeterminacy of ARG profiles in chemically fertilized soils compared to organically fertilized soils (Liu W. et al., 2022). Studies have indicated that the application of nitrogen-phosphorus-potassium (NPK) fertilizers can lead to significant alterations in bacterial communities, while having a moderate impact on ARG diversity and abundance (Liu et al., 2017; Xie et al., 2018b; Wang F. et al., 2020). Furthermore, research has shown that the effects of chemical fertilizers on ARGs vary depending on soil type, with an increase in relative abundance observed in dryland soil and a decrease in paddy soil (Wang F. et al., 2018). In addition to these, numerous academic studies have demonstrated that the use of chemical fertilizers can increase the presence of ARGs and antibiotic-resistant pathogens in soil (Sun et al., 2019; Huang et al., 2021; Yang et al., 2022), thereby posing a potential threat to human health through plant endosphere transmission. Research has also suggested that N fertilization may be a more effective method than PK fertilizers in mitigating the risks of ARGs in soil exposed to reclaimed water irrigation (Cui et al., 2022). Additionally, scholars have highlighted the close association between ARGs and various N-cycling-related genes (NCRGs) (Wang M. et al., 2020), suggesting that potential hosts for ARGs may coexist with hosts for nitrate reduction genes (NRGs) (Hu et al., 2022). Moreover, the enforcement of denitrification has been found to contribute to a decrease in antibiotic and ARG levels (Sun et al., 2017; Wang L. et al., 2022), underscoring the importance of nitrogen fertilization in regulating soil ARGs.

The forms of nitrogen present have been identified as a factor influencing ARG profiles in both soil and plants. Sun et al. have demonstrated that the application of NH_4_^+^–N and NO_3_^−^–N may stimulate the abundances of ARGs in soil without significant differences (Sun et al., 2020). A study demonstrated that the positive impact of NH_4_^+^–N accumulation in soil was more pronounced than that of NO_3_^−^–N, although this trend was not observed in plants (Wang T. et al., 2022). A recent study has indicated that the concentration of NH_4_^+^–N may serve as a significant determinant of ARG profiles in soil (Wang et al., 2024). However, previous research has indicated that NRGs and ARGs coexist, suggesting that NO_3_^−^–N may exert a greater influence on promoting ARG abundances compared to NH_4_^+^–N (Hu et al., 2022). These differences may be attributable to the varied soil management practices employed during the field experiment. For instance, NH_4_^+^–N generally exhibits greater stability when associated with soil particles compared to NO_3_^−^–N (Zhuang, 1999), which could influence the concentrations of different nitrogen forms and the microbial utilization processes within the soil (Peacock et al., 2001). These factors are likely to exert differential effects on the soil physical and chemical properties, as well as on soil microbial communities (de Nijs et al., 2019; Alster et al., 2020), thereby ultimately impacting ARGs (Forsberg et al., 2014; Kang et al., 2018a). Therefore, to address potential variability in the field experiment, it is essential to conduct a controlled laboratory experiment to investigate possible alterations in ARG profiles resulting from different nitrogen sources.

The current study utilized metagenomic technology to assess the effects of three nitrogen sources, along with a control group, on soil ARGs over time. This research aimed to examine three principal objectives: (i) the potential variances in core ARGs profiles resulting from various forms of nitrogen application, (ii) the relationship between core ARGs and NCRGs, and (iii) potential mechanisms that may explain the divergent profiles of core ARGs. This research offers significant insights for the advancement of sustainable agricultural practices and environmental management strategies.

## Materials and methods

### Soil sampling

On March 17, 2023, soil samples were collected from a weed-infested area on the campus of Yancheng Teachers University, where no fertilization had occurred for a minimum of 3 years. The sandy loam soil exhibited characteristics such as a pH of 8.60 (1:1 soil to water ratio), 9.36 g/kg of organic matter, 2.89 mg/kg of ammonia nitrogen (NH_4_–N), and 25.00 mg/kg of nitrate nitrogen (NO_3_^−^–N). Prior to analysis, the soil was air-dried and passed through a 2 mm sieve.

### Experimental design

A total of 36 Petri dishes with a diameter of 150 mm were prepared and filled with 40 g of soil each. Concentrated stocks of NaNO_3_, NH_4_Cl, and CO(NH_2_)_2_ were previously prepared separately and evenly added to three randomly selected dishes (replicates) at a final concentration of 100 mg N/kg. Additionally, nine of the 36 Petri dishes containing the same soils were supplemented with equal volumes of water as a reference to the N fertilization treatments. Therefore, four treatments were established: CK (no N addition), nitrate (NN), ammonia (AN), and urea (ON) treatments.

The Petri dishes were incubated at 25°C with 60% humidity for a period of 30 d. On days 0, 15, and 30 post-fertilization, approximately 1.0 g of mixed soils from three randomly selected points within each Petri dish were collected and preserved at −80°C. To minimize sampling-related disturbances, nine replicates were established for each treatment, with three of these replicates being randomly selected for sampling at varying time intervals.

### Metagenomic sequencing and ARGs analysis

Microbial DNA was extracted from soil samples using a PowerSoil® DNA Isolation Kit and verified through agarose gel electrophoresis. Fragmentation of the DNA was achieved with a Covaris M220 sonicator, with selection of approximately 450 bp fragments for library construction. Subsequently, a paired-end library was generated utilizing the TruSeq PE Cluster Kit v3-cBot-HS and TruSeq SBS kit v.3-HS sequencing kit from Illumina. Alkali degeneration was employed to produce single-stranded DNA fragments. The treated samples underwent sequencing using an Illumina NovaSeq 6000 platform at Shanghai BIOZERON Biotechnology Co., Ltd. Raw sequences that were contaminated by adapters or contained more than 10% unknown nucleotides were excluded. Additionally, reads with abnormal nucleotides at the 5′ ends and those shorter than 75 base pairs after quality control were eliminated.

The resulting clean sequences were analyzed for ARGs using the ARG analysis pipeline (ARG-OAP, v2.2) with cutoff parameters of *e-*value ≤10^−7^, sequence identity ≥80%, and alignment length ≥25 amino acids (Yin et al., 2018). Reads were aligned against the Structured Antibiotic Resistance Genes (SARG, v2.2) and BacMet databases using BLASTX with recommended parameters, resulting in the annotation of reads as either ARG or metal resistance genes (MRG) (Ma et al., 2017). ARG types and subtypes were automatically identified using ARGs-OAP (v2.0), and their relative abundances were quantified as “ARG copy per cell” based on a specific equation (Jia et al., 2020; Yin et al., 2023):


$$\mathrm{Abundance}=\overset{n}{\underset{i=1}{\sum }}\frac{N_{ARG-\mathrm{like reads}}\times L_{\mathrm{read}}/L_{ARG\;\mathrm{reference sequence}}}{\mathrm{Cell number}}$$

Where, N_ARG-like reads_ represents the number of the ARG-like sequences matched with one specific ARG reference sequence; L_read_ is the read length (150 bp); L_ARG reference sequence_ is the nucleotide sequence length of the correspondingly specific ARG reference sequence (bp); Cell number is the estimated number of bacterial cells in each metagenomic dataset calculated by ARGs-OAP based on the searching results of 30 sets of essential single copy marker genes (Yin et al., 2018); n is the number of mapped ARG reference sequences belonging to the target ARG types/subtypes. Besides, the percentage (%) of individual ARG type/subtype was calculated as the ratio of the relative abundance of target ARG type/subtype versus the relative abundance of all ARGs in each sample. Furthermore, the trimmed reads underwent taxonomic classification using the Kraken2 (v2.0.6) database, which includes the NCBI reference nucleotide database (RefSeq) for various taxonomic levels (Wood and Salzberg, 2014).

### Data analysis

In this study, the ARGs and mobile genetic elements (MGEs) were identified as core ARGs and core MGEs, defined as persistent ARGs present in all sites regardless of treatment over time. The raw data was analyzed using SPSS Statistics for Windows version 18.0 to calculate means and standard errors. A two-way analysis of variance (ANOVA) was conducted to examine the impact of nitrogen forms (NN, AN, and ON) and time (0 d, 15 d, and 30 d) on the levels of ARGs in different treatments. Subsequently, Bonferroni’s post-hoc tests were conducted to assess significant differences among treatments within a group corresponding to the x-axis at a significance level of 0.05. Principal component analysis (PCA) was utilized to analyze the relative abundance of ARGs and heatmaps were generated using the ClustVis online tool (Metsalu and Vilo, 2015). The column and line charts featured in this study were produced using Sigma Plot for Windows Version 10.0 (Systat Software, San Jose, CA). Furthermore, to investigate the potential correlation between core ARGs and NCRGs, NCRGs were annotated utilizing the integrative database NCycDB with an e-value cutoff of 10^−7^ (Tu et al., 2019).

## Results

### Profiles of core ARGs following N fertilizations over time

**Figure 1:**
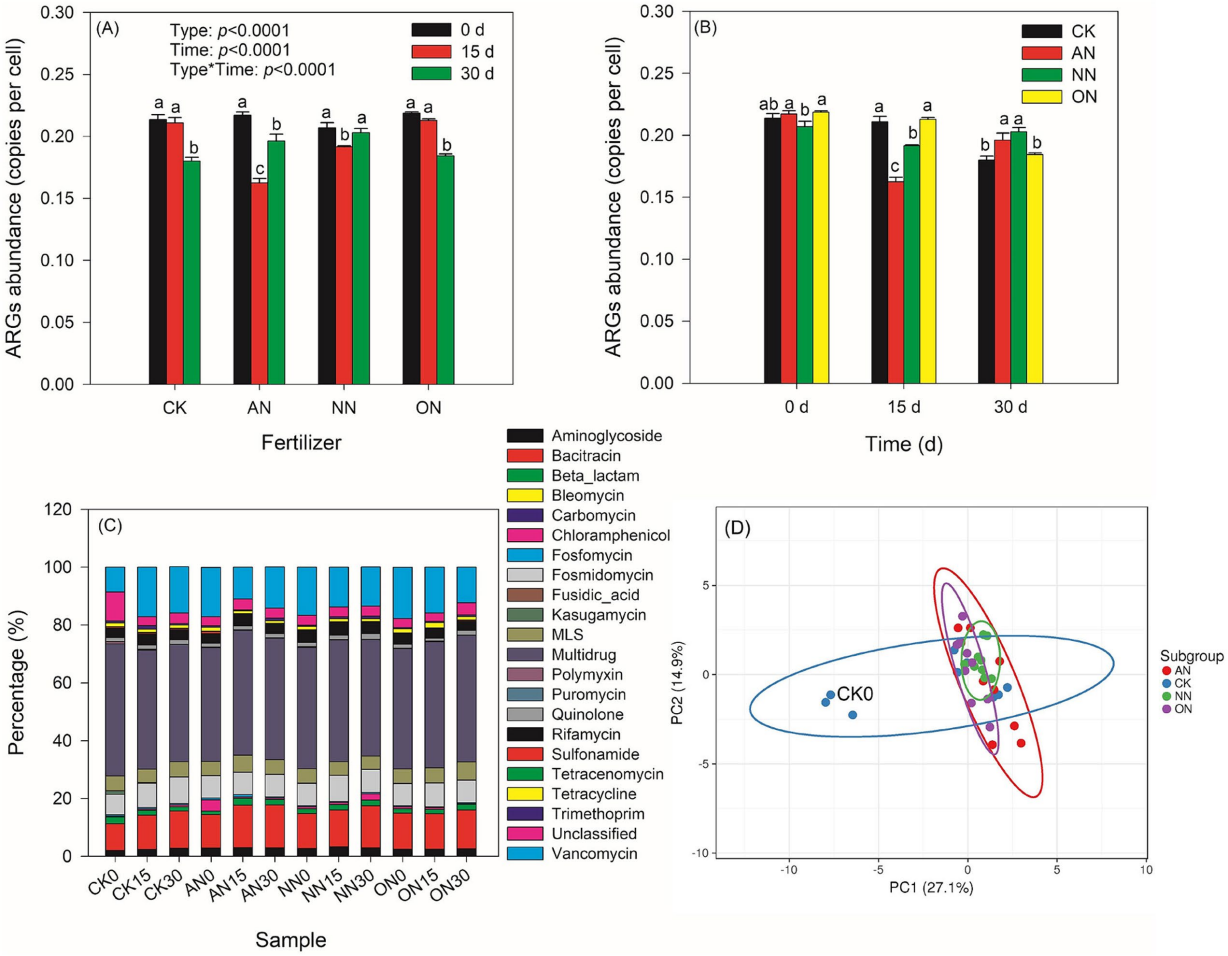
Effects of different chemical N fertilizers on core ARGs profiles with time in soil. Chemical N fertilizers have limited impacts on core ARGs abundances **(A,B)**. Similar compositions of ARGs among treatments with time **(C)**. Principal component analysis (PCA) shows that all the treatments can be categorized together except CK0, which is labelled in the panel **(D)**. In panels **(A,B)** different letters represent significant differences within one group corresponding to the x-axis.

A total of 22 types and 381 subtypes of ARGs were identified. Among these, 101 core ARGs were consistently present in all treatments over time and were used for analysis. The abundance of ARGs in all treatments ranged from 0.16 to 0.22 copies per cell (Figure 1A). The trend of ARG abundance in the treatment with nitrogen fertilization was similar to that in the control group, and there was no significant difference between the two groups at the same sampling time (Figure 1B). Fertilizations with AN and NN have a significant impact on the abundance of ARGs over a 30-day period. Specifically, there is a notable decrease in ARG abundance on day 15 following fertilization with AN and NN, with a subsequent increase on day 30. The abundance of ARGs in the AN treatment on day 15 is significantly lower than that in the NN treatment (Figure 1B), although there is no statistical difference between the two treatments on day 30. Additionally, the changes in MGEs following nitrogen fertilizations are more pronounced than those in core ARGs (Supplementary Figures S1A,B). Nitrogen fertilizations lead to a decrease in MGE abundance on day 30 compared to day 0. MGE abundance in the ON treatment is lowest on both day 0 and day 30, while the AN treatment shows the lowest abundance on day 15.

The prevalence of unclassified ARGs is significantly higher in the CK0 treatment compared to other treatments (Figure 1C). Notably, the percentages of Bacitracin and Vancomycin ARGs exhibit distinct differences among the treatments. PCA demonstrates that all treatments, except for CK0, exhibit similar ARG profiles (Figure 1D). CK treatment displays more pronounced changes over time compared to N fertilization treatments, which show convergent ARG profiles, particularly evident in the NN treatment. These findings are further supported by PCA analyses conducted over time and across treatments. These findings can be further confirmed through PCA over time or across different treatments. The lower explanations of PC1 in the N fertilization treatments compared to the CK (Supplementary Figures S2A–D) suggest that N fertilization leads to more consistent profiles of ARGs over time. Additionally, the decreasing explanation values of PC1 over time (Supplementary Figures S2E–H) indicate that time plays a crucial role in the convergence effects of N fertilization.

### Potential bacterial hosts for ARGs

**Figure 2:**
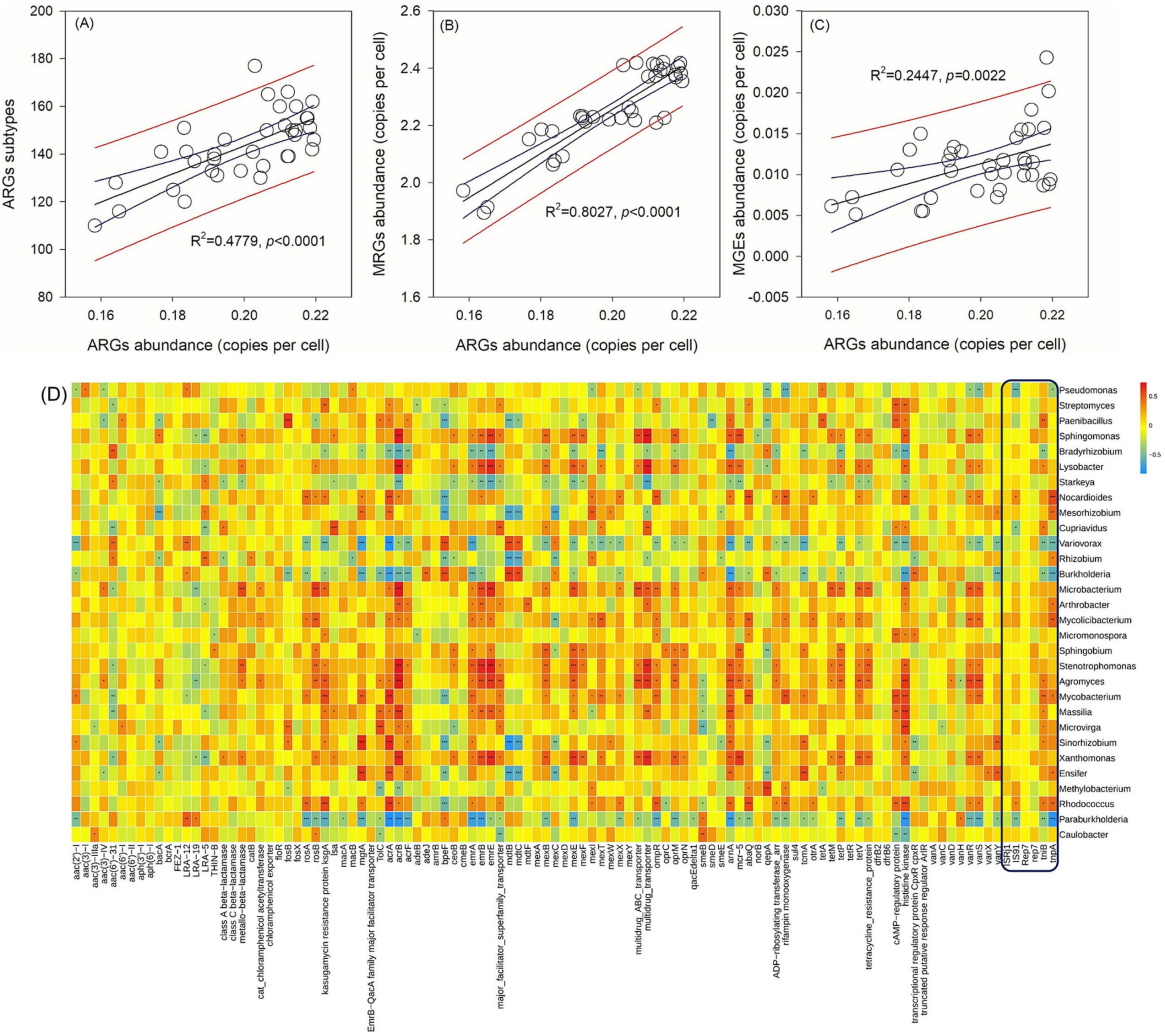
Correlations between core ARGs abundances and ARGs subtypes **(A)**, core MRGs abundances **(B)**, and core MGEs abundances **(C)**. A correlation matrix between ARGs and bacterial genera **(D)**. Different numbers of asterisk (*, **, ***) in panel **(D)** represent significant correlations using the Pearson’s correlation coefficient at *p*-values of 0.05, 0.01, and 0.001.

**Figure 3:**
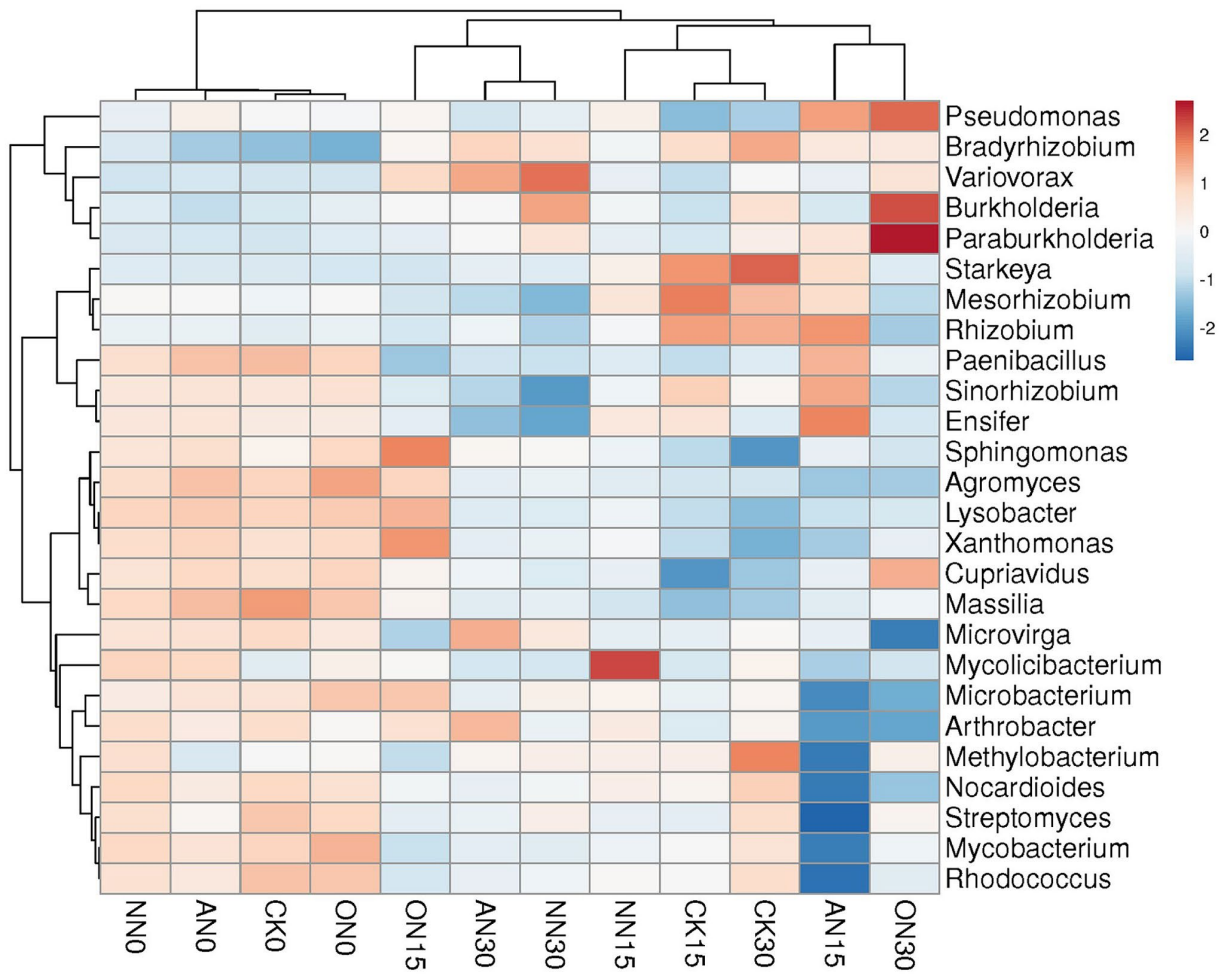
The heatmap of the potential bacterial hosts for core ARGs.

The abundance of ARGs exhibits a positive correlation with the quantities of ARG subtypes (Figure 2A), metal resistance genes (MRGs) (Figure 2B), and MGEs (Figure 2C). MGEs play a crucial role in facilitating the horizontal transfer of ARGs among bacterial strains. Consequently, the identification of bacterial species that demonstrate positive correlations with both ARGs and MGEs serves as a key criterion for screening potential hosts in this study. Specifically, among the six core MGEs examined, *IS91*, *tniB*, and *tnpA* exhibit positive correlations with certain bacterial species (Figure 2D). According to the aforementioned criteria, *Paenibacillus*, *Lysobacter*, *Nocardioides*, *Mesorhizobium*, *Cupriavidus*, *Rhizobium*, *Arthrobacter*, *Mycolicibacterium*, *Agromyces*, *Mycobacterium*, *Massilia*, *Microvirga*, *Sinorhizobium*, *Ensifer*, and *Rhodococcus* are considered potential bacterial hosts. These bacteria may harbor multiple ARGs within a single cell. For instance, *Rhodococcus* has been found to contain *rosA*, *ksgA*, *mgtA*, *acrA*, and other ARGs simultaneously. Among these potential hosts, *Paenibacillus* and *Massilia* are identified as potential human gut microbes based on a search of the Human Gut Microbiome Database (hGMB),^1^ and *Mycobacterium* and *Rhodococcus* are considered potential clinical pathogens based on searches conducted using GlobalRPh.^2^ Furthermore, the results of the clustering analysis indicate that the potential bacterial hosts can be grouped based on time rather than fertilizer types (Figure 3). Additionally, there are no discernible distinctions in potential hosts between days 15 and 30.

### Changes in bacterial communities following N fertilizations with time

**Figure 4:**
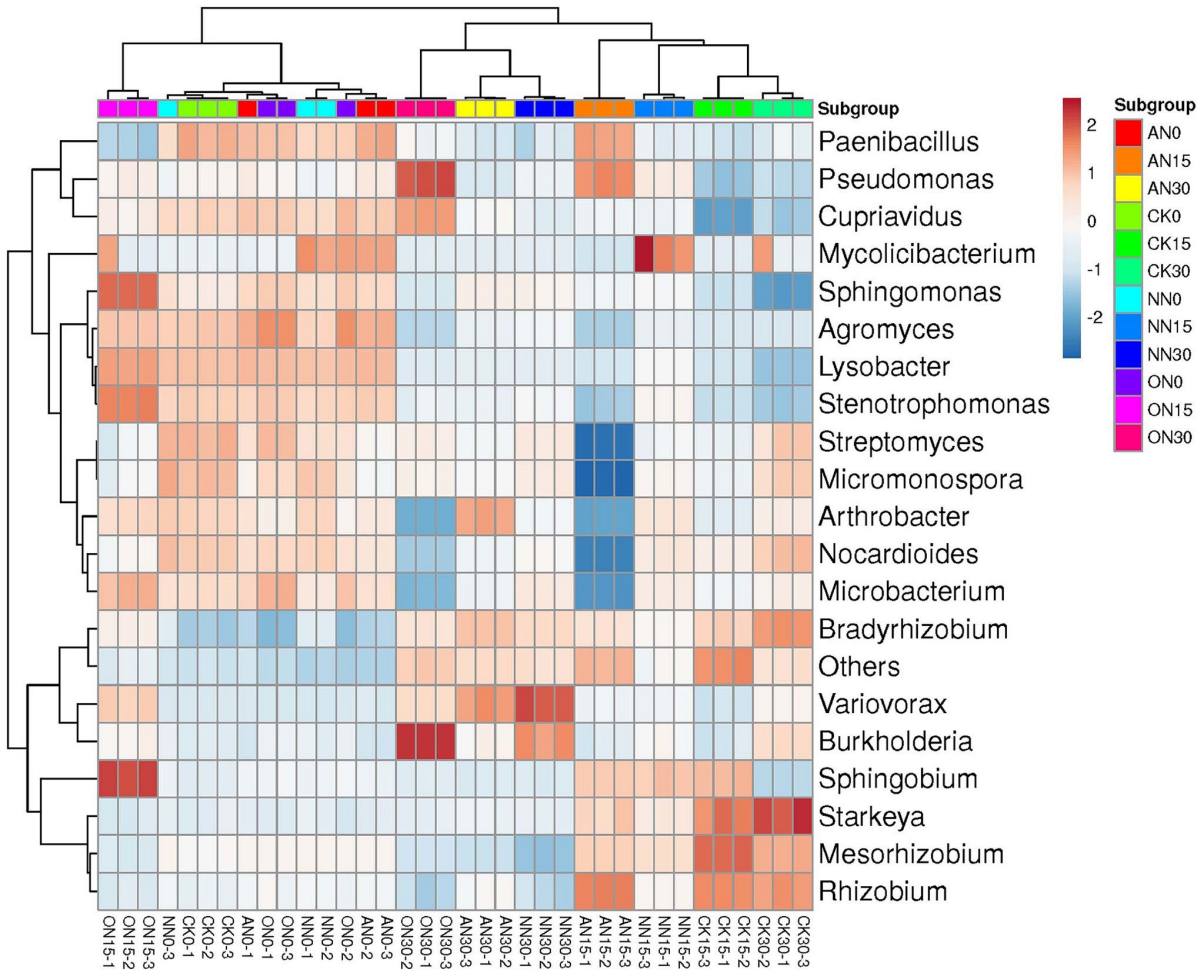
The heatmap of bacterial community using top 20 abundant genera. “Others” represents the remaining sum of genera.

The bacterial communities across treatments can also be classified by time, with the exception of ON15 and CK30, which are grouped with those on day 0 and day 15, respectively (Figure 4). The upper left taxa, including *Paenibacillus*, *Pseudomonas*, and *Cupriavidus*, exhibit high abundance on day 0, which subsequently decreases over time (Figure 4). Conversely, *Bradyrhizobium*, Var*iovorax*, and *Burkholderia* demonstrate relatively high abundance on day 30. The ON30 treatment displays the highest abundances of *Burkholderia* and *Pseudomonas*, while *Variovorax* is more abundant in NN30 and AN30. *Starkeya* emerges as the most abundant species in CK30. PCA reveals that all treatments can be clearly distinguished by time, with high explanatory values of PC1, albeit without significant differences (Supplementary Figures S4A–D). In contrast to ARGs, various nitrogen fertilizers have a significant impact on bacterial communities. The explanatory power of PC1 increases from 39.5% on day 0 (Supplementary Figure S3E) to 63.6% on day 15, allowing for clear differentiation of treatments through PCA (Supplementary Figure S3F). However, there is a slight decrease in explanatory power on day 30, where the AN and NN treatments are indistinguishable compared to day 15 (Supplementary Figure S3H). These findings suggest that both temporal factors and the type of fertilizer utilized can have a substantial influence on bacterial communities.

### The relationship of ARGs and NCRGs in soil

**Figure 5:**
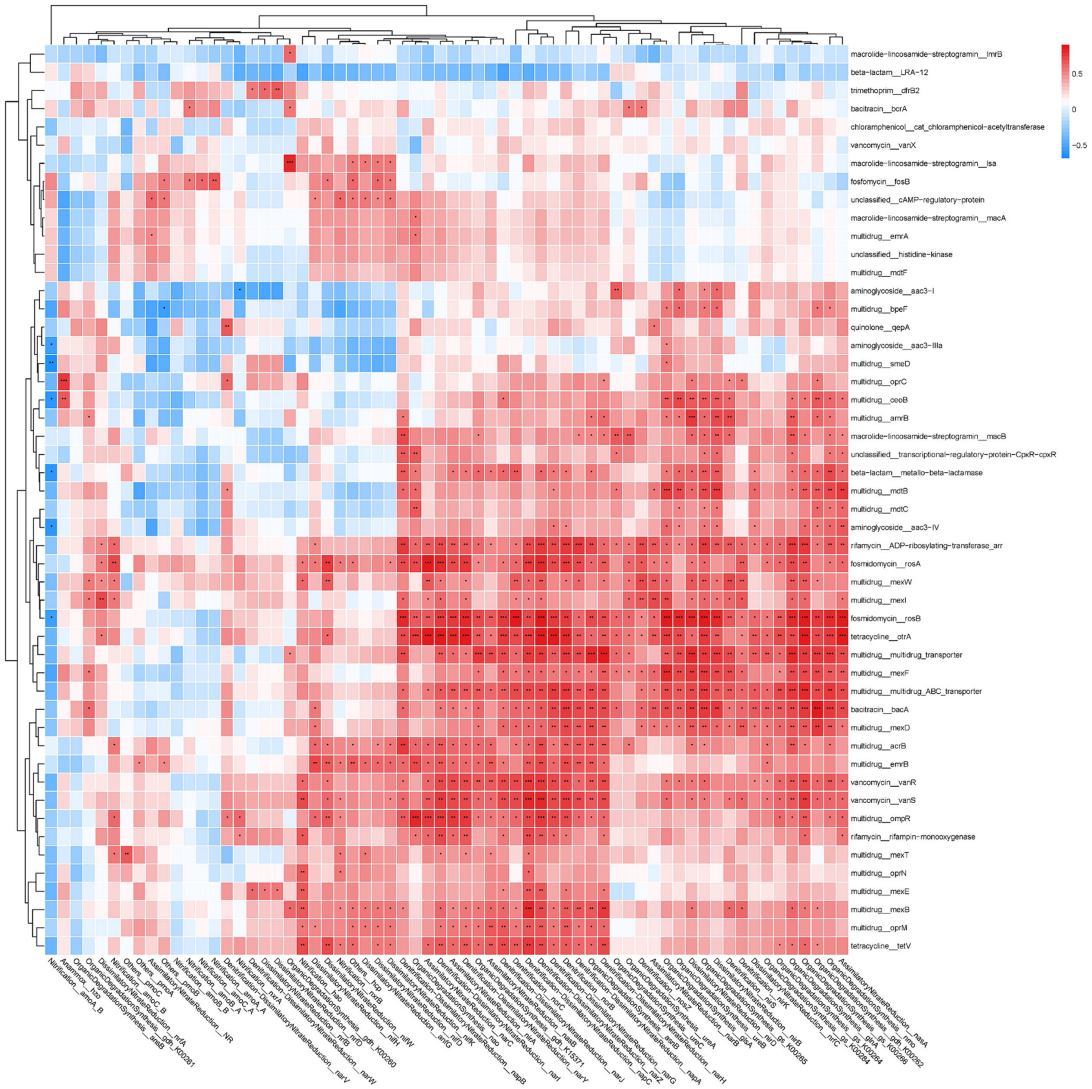
The correlation matrix between ARGs and nitrogen-cycling related genes (NCRGs). Different numbers of asterisk (*, **, ***) represent significant correlations using the Pearson’s correlation coefficient at *p*-values of 0.05, 0.01, and 0.001.

Most NCRGs, particularly NRGs, exhibit a significant correlation with ARGs (Figure 5). Within the classification of NRGs, two principal categories can be distinguished: nitrogen-fixing genes, such as *nifK*, *nifD*, and *nifH*, and denitrification genes, including *narG*, *napA*, *nirK*, *norB*, and *nosZ*. Notably, several ARGs such as *rosA*, *rosB otrA*, *mexF*, *bacA*, and *vanS* are found to be correlated with these NRGs. In order to assess reliability, two representative strains of potential bacterial hosts listed in Figure 3, specifically *Pseudomonas aeruginosa PAO1* (NCBI Taxonomy ID 287) and *Burkholderia pseudomallei* (NCBI Taxonomy ID 28450), were examined in the Genome database of the National Center for Biotechnology Information (NCBI)^3^ following the retrieval of their annotated genomes. Analysis revealed the presence of ARGs *mexB*, *oprM*, *bacA*, *mexF*, *mexD* and NRGs *napA*, *narJ*, *narH*, *narG* within *P. aeruginosa* PAO1, all of which are documented in Figure 5. Similarly, *emrB*, *bacA* and *narJ*, *narH*, and *narG* were identified in *B. pseudomallei.*

## Discussion

### Limited impact of N fertilization on the core ARGs profile in soil

There is ongoing debate regarding the effects of chemical fertilizer application on soil ARGs. A study demonstrated that a 15-year application of chemical fertilizers did not have a significant impact on the relative abundance of total ARGs (Wang F. et al., 2020). Additionally, a study showed that single nitrogen treatment reduced bacterial diversity and abundance, leading to the elimination of ARGs (Cui et al., 2022). However, other studies have suggested that N fertilizers can have a strong influence on the content of soil ARGs (Forsberg et al., 2014). A study demonstrated that the application of chemical fertilizer resulted in an increase in the relative abundance of ARGs in dryland soil, while decreasing their relative abundance in paddy soil (Wang F. et al., 2018). A recent studies employing metagenomic technology demonstrated that nitrogen limitation predominantly facilitated the dissemination of ARGs through HGT events (Liu et al., 2024). However, another study reported a contrasting result (Wang et al., 2024). These findings indicate that additional factors, beyond nitrogen fertilizers, may play crucial roles in the regulation of ARGs. Therefore, a control experiment is necessary to investigate the potential effects of nitrogen fertilizers on soil ARGs. The findings of a microcosmic experiment presented in this study reveal that chemical N fertilizers have varying impacts on the abundances of ARGs and MGEs, depending on their forms and the timing of sampling in the short term (30 days). While there were increases in the abundance of ARGs in treatments with AN and NN on day 30, these increases were not significantly higher compared to the CK on day 0. Overall, the application of chemical N fertilizers has limited effects on the accumulation of ARGs in soil. This finding is consistent with the composition of ARGs as reflected in PCA.

Numerous studies have indicated that the accumulation of ARGs in soil is primarily attributed to the regulation of bacterial communities by environmental factors, such as fertilizers (Liu et al., 2017; Han et al., 2018; Wang M. et al., 2018). Our research revealed that varying levels of nitrogen fertilization can lead to significant shifts in bacterial communities within a short period of 15 d, yet only have minimal effects on the abundance of ARGs. It is suggested that exogenous additives lacking ARGs and/or not participating in co-occurrence events may have limited influence on the levels of ARGs in soil. The nitrogen input rate utilized in this study was 100 mg/kg, equivalent to 2,250 kg N/ha. In the natural environment, nitrogen deposition can reach levels of up to 90 kg N/ha/year (Verma and Sagar, 2020; He et al., 2021), requiring a period of 25 years of continuous N deposition to match the rate of addition in the absence of additional N input. Previous research has indicated minimal fluctuations in ARGs during the soil maturation process over an 86-year period (Tang et al., 2021). Consequently, it is suggested that a 25-year period of N deposition may not lead to substantial variations in ARGs within the soil, potentially explaining the limited impacts observed in the present study.

### Effects of N forms on ARGs profiles

Soil microbes exhibit a preference for NH_4_^+^–N over NO_3_^−^–N, contingent upon the specific types of carbon sources introduced (Romero et al., 2015). Contrary to previous findings, a study indicated that soil microbes did not exhibit a preference for either form of nitrogen (Harrison et al., 2007). NH_4_^+^–N levels experienced a rapid decline following application, stabilizing by day 15 across all treatments (Supplementary Figure S4A). Conversely, NO_3_^−^–N levels consistently remained highest in the NN treatment over time, persisting at elevated levels in both NN and ON treatments (Supplementary Figure S4B). This finding suggests that organisms with a preference for NH_4_^+^–N over NO_3_^−^–N are dominant species in this environment, likely due to limited available carbon in the soil. For instance, the prevalent species in this investigation (Romero et al., 2015), such as *Pseudomonas* (Zhang et al., 2019), *Rhizobium* (Ye et al., 2021), and *Sphingomonas* (Banik et al., 2007) have been shown to favor NH_4_^+^–N over NO_3_^−^–N. Therefore, the preference for N forms may contribute to significant alterations in the bacterial community. Despite this shift, there was no corresponding change in ARGs profiles. Generally, microbes exhibit a preference for inorganic N uptake over organic N in most instances (Moe, 2013). In the present investigation, the inclusion of urea yielded comparable outcomes to CK in terms of the abundance of ARGs, suggesting that urea may be a more environmentally benign N fertilizer option when compared to AN and NN. Moreover, given the relatively high N content and eco-friendly manufacturing process associated with CO_2_ fixation (Alper and Yuksel Orhan, 2017), controlled-release urea may serve as a viable chemical N fertilizer for agricultural applications (Tian et al., 2021).

The influence of nitrogen fertilizers on the abundance of ARGs exhibited variability. A study employing real-time quantitative PCR (qPCR) demonstrated that distinct forms of nitrogen (NH_4_^+^–N and NO_3_^−^–N) could affect ARG abundance; however, these forms were not identified as primary determinants (Wang T. et al., 2022). In a separate study, it was found that NH_4_^+^–N significantly increased ARG abundance using qPCR (Sun et al., 2020). Conversely, studies suggesting that chemical fertilizers had minimal effects on ARGs employed high-throughput quantitative PCR or metagenomic sequencing technologies (Xie et al., 2018b; Wang F. et al., 2020). Therefore, the variation in outcomes may be attributed to the divergent methodologies utilized. Conventional qPCR serves as an effective tool for promptly identifying and quantifying ARGs, facilitating the comparative analysis of antibiotic resistance, particularly for specific ARGs like tetracycline resistance genes (Su et al., 2017; Kang et al., 2022). However, this also presents a limitation of qPCR in terms of its restricted target detection capabilities. Besides, Due to the high diversity of DNA templates and complexity in environmental samples, the use of qPCR may lead to the overestimation or even false-positive detection of target genes, attributable to potential non-specific amplification (Li and Yan, 2021). By contrast, metagenomics is proposed as an effective tool for conducting a thorough investigation of environmental ARGs (Liu et al., 2019). Besides, it is strongly recommended that results be compared using consistent methodologies. For example, the abundance of the key MGE *intI1* is often quantitatively assessed due to its potential involvement in HGT (Liu X. et al., 2022). However, other essential MGEs such as *tnpA*, *tniB*, and *IS91* exhibit a higher co-occurrence with ARGs compared to *intI1* in this study, consistent with previous findings (Li et al., 2020; Zhao et al., 2021). Such patterns can only be elucidated through the use of high-throughput technologies.

### The relationship of ARGs and NCRGs in soil

Currently, there is a limited number of academic papers discussing the potential co-occurrence of ARGs with NCRGs. Previous studies have demonstrated positive correlations between the ratio of AOA/AOB *amoA* and soil available Cd and Cu contents (Li et al., 2009; Zhang et al., 2017). Furthermore, research has indicated that most ARGs, MRGs, and NRGs share the same host bacterial species in leachates (Wang et al., 2021). The co-occurrence of ARGs and MRGs is a common phenomenon, which is also reflected in the close relationship between ARGs and MRGs observed in this study. Our findings further demonstrated that numerous NCRGs (especially NRGs) exhibit a positive correlation with ARGs, which again suggests the existence of close relationships among ARGs, MRGs, and NRGs. A study demonstrated that a significant percentage (75.3–94.9%) of microorganisms carrying ARGs also possess NRGs, suggesting that nitrate reducing bacteria may serve as primary hosts for ARGs (Hu et al., 2022). Other studies have concluded that ARGs exhibit positive correlations with denitrification processes (Wu et al., 2017; Rahman et al., 2018; Wang et al., 2024). However, a study revealed a negative correlation between NRGs and ARG abundance (Sun et al., 2017). This discrepancy may be due to variations in methodology, as the use of qPCR for gene detection can yield differing results. Our previous research demonstrated a higher prevalence of tetracycline resistance genes in wet soils compared to dry soils (Kang et al., 2018a). It is established that denitrification processes are augmented in wet soils owing to elevated levels of NRGs (Sun et al., 2017), implying a positive correlation between ARGs and NRGs. A study confirmed that the moisture content of soil has an impact on the bioavailability of stress factors such as metals and antibiotics, leading to increased pressure in soil niches (Markowicz et al., 2021). Consequently, it is suggested that ARGs may enable denitrifying bacteria to better adapt to more challenging environments.

## Conclusion

The prevalence of core ARGs in ON treatment shows similarity to CK treatment over time, suggesting that ON treatment, particularly in a controlled release dosage form, may serve as an environmentally sustainable N fertilizer. While chemical N fertilizers have minimal effects on the core ARGs profile as determined by PCA and clustering analyses, different N fertilizers can induce significant and distinct changes in bacterial communities. Nevertheless, the core ARGs’ potential bacterial hosts remain unaffected by N fertilizers, which may explain the limited impact on the core ARGs profile. The presence of nitrogen-fixing genes and denitrification genes has been found to be positively associated with ARGs such as *rosA*, *mexF*, *bacA*, *vanS*, etc., suggesting a potential risk of ARG proliferation in environments with high denitrification activity. Concerns regarding the accumulation of ARGs due to the use of chemical N fertilizers should be addressed.

## Data Availability

The original contributions presented in the study are included in the article/Supplementary material, further inquiries can be directed to the corresponding author.

## Footnotes

^1^https://hgmb.nmdc.cn/

^2^https://globalrph.com/

^3^https://www.ncbi.nlm.nih.gov/genome/
