# Supplementary material for: Applications of different forms of nitrogen fertilizers affect soil bacterial community but not core ARGs profile
Source: Front Microbiol. 2024 Oct 1;15:1447782. doi: 10.3389/fmicb.2024.1447782 (PMC11480956; doi:10.3389/fmicb.2024.1447782)
Supplement: Supplementary file 1 [file Data_Sheet_1.docx]

Fig. S1





Fig. S2


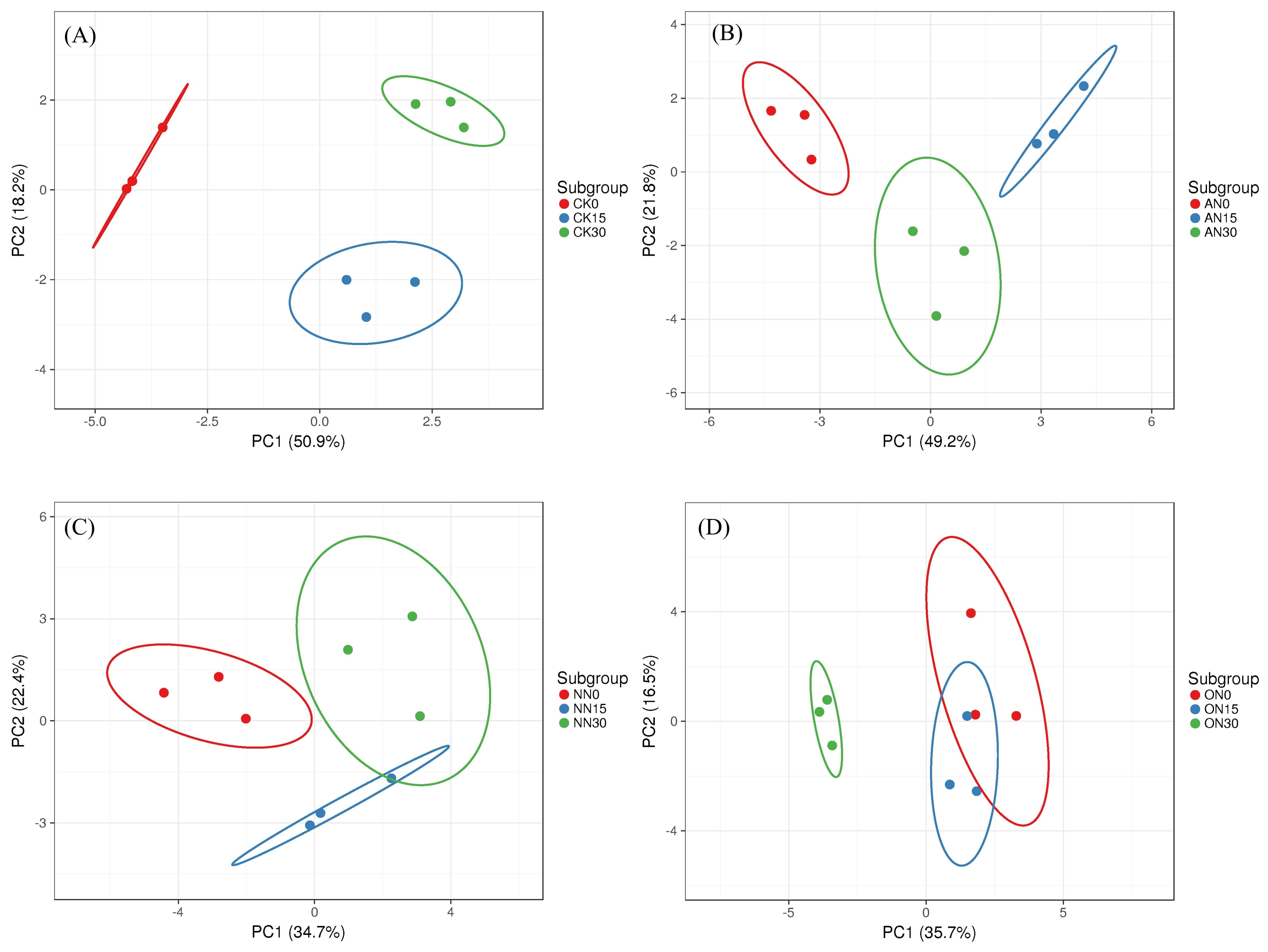


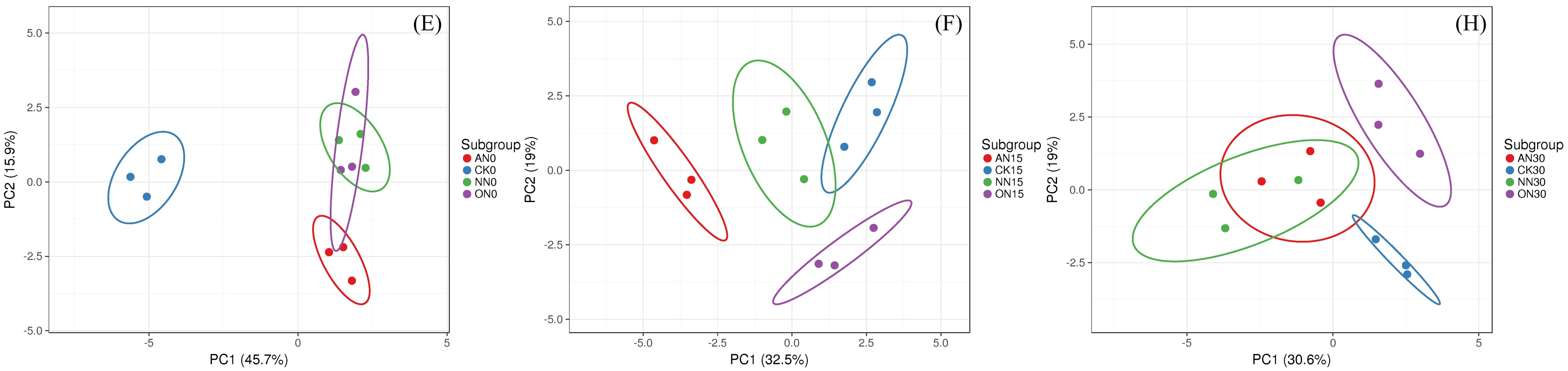


Fig. S3


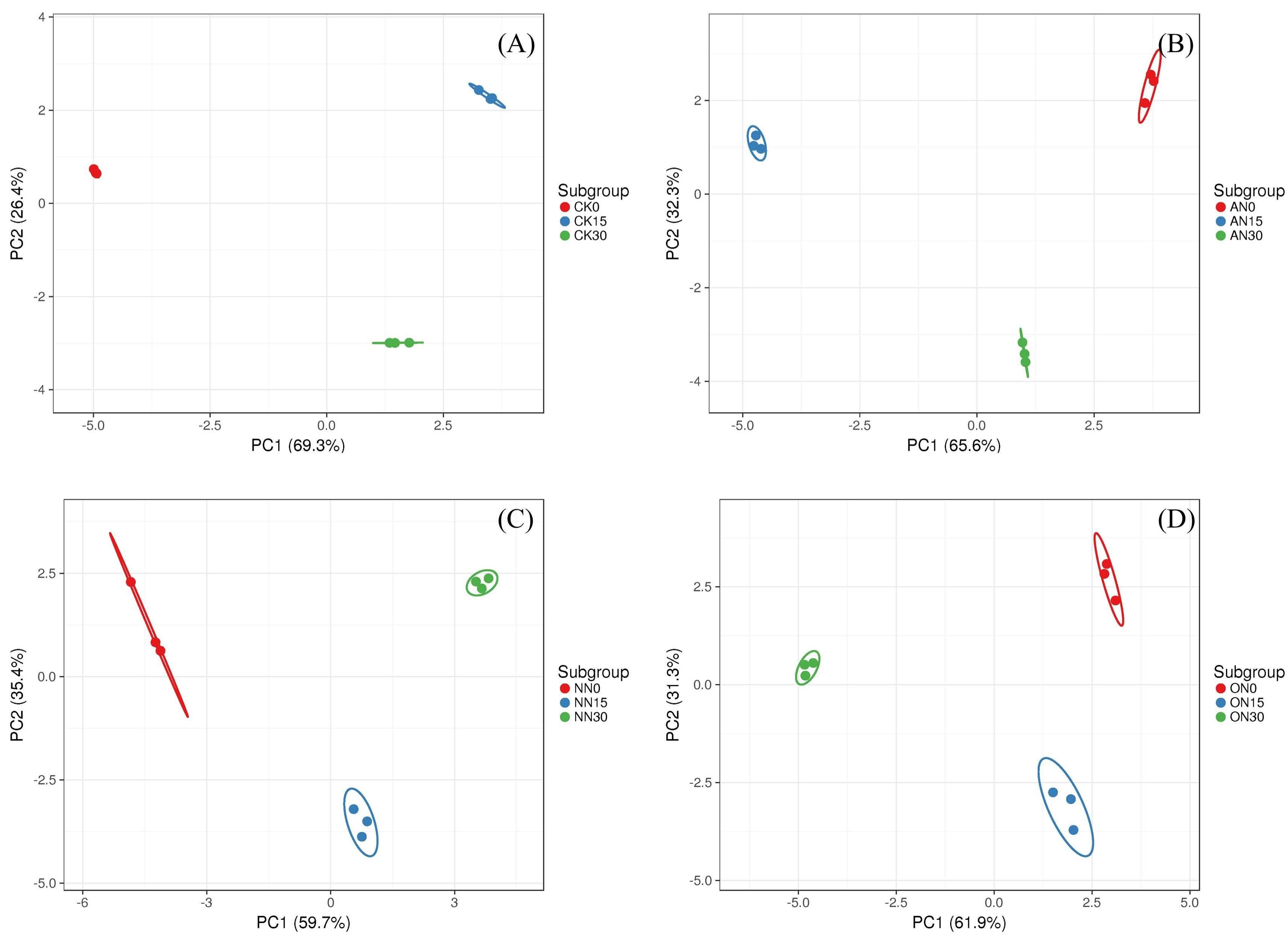


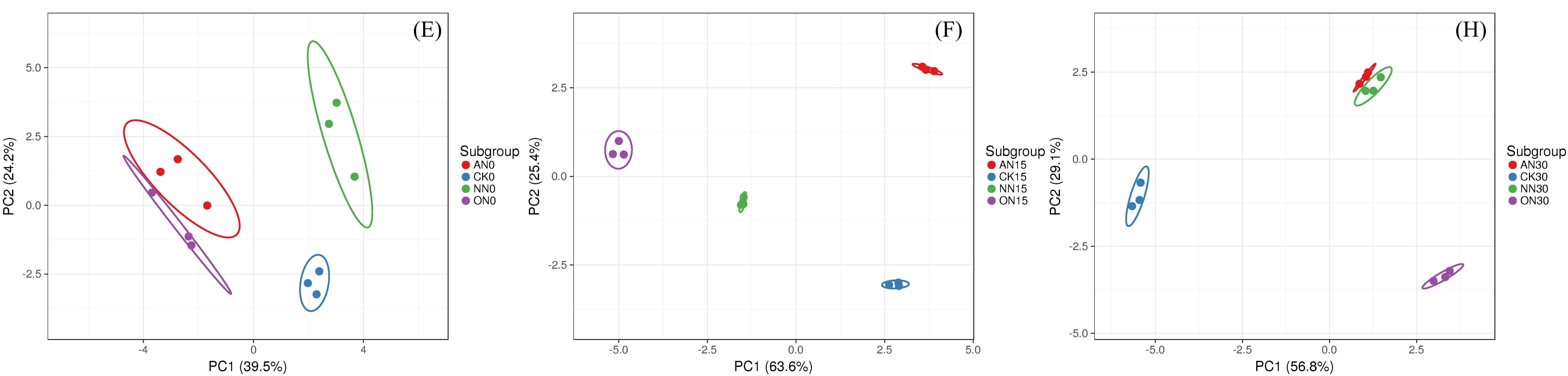


Fig. S4


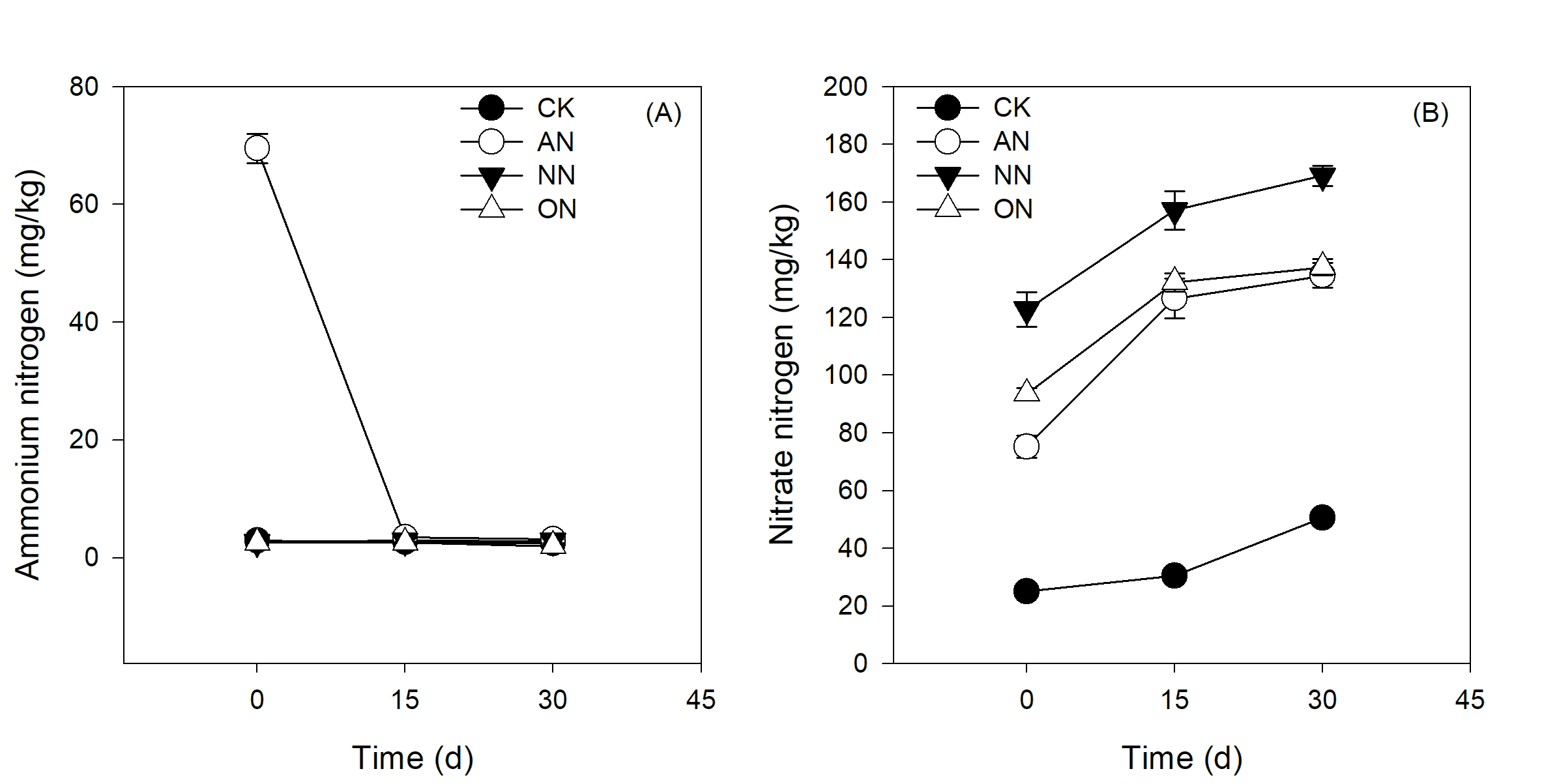


**Figure legends:**

Fig. S1 Effects of different chemical N fertilizers on core MGEs abundances with time in soil. Panels A and B are exhibited with different arrangements of the same data. Different letters represent significant differences within one group corresponding to the x-axis.

Fig. S2 Comparisons of changes in core ARGs profiles with time (A-D) and among treatments (E-H) by principal component analysis (PCA) based on Bray-Curtis distances.

Fig. S3 Comparisons of changes in bacterial communities’ profiles with time (A-D) and among treatments (E-H) by principal component analysis (PCA) based on Bray-Curtis distances.

Fig. S4 Time courses of the contents of ammonium-N (A) and nitrate-N (B).
